# Systematic evaluation of the prognostic and immunological role of PDLIM2 across 33 cancer types

**DOI:** 10.1038/s41598-022-05987-1

**Published:** 2022-02-04

**Authors:** Yudan Zeng, Dongtao Lin, Mengqian Gao, Guoxia Du, Yongming Cai

**Affiliations:** 1 Guangdong Pharmaceutical University, Guangzhou, China; 2 Key Specialty of Clinical Pharmacy, The First Affiliated Hospital of Guangdong Pharmaceutical University, Guangzhou, China; 3 Guangdong Provincial TCM Precision Medicine Big Data Engineering Technology Research Center, Guangzhou, China; 4 NMPA Key Laboratory for Technology Research and Evaluation of Pharmacovigilance, Guangzhou, China

**Keywords:** Computational biology and bioinformatics, Data mining, Protein analysis

## Abstract

The protein PDLIM2 regulates the stability of various transcription factors and is required for polarized cell migration. However, the clinical relevance and immune infiltration of PDLIM2 in cancer are not well-understood. We utilized The Cancer Genome Atlas and Genotype-Tissue Expression database to characterize alterations in PDLIM2 in pan-cancer. TIMER was used to explore PDLIM2 expression and immune infiltration levels. We assessed the correlation between PDLIM2 expression and immune-associated gene expression, immune score, tumor mutation burden, and DNA microsatellite instability. PDLIM2 significantly affected the prognosis of various cancers. Increased expression of PDLIM2 was significantly correlated with the tumor grade in seven types of tumors. The expression level of PDLIM2 was positively correlated with immune infiltrates, including B cells, CD8+ T cells, CD4+ T cells, neutrophils, macrophages, and dendritic cells in bladder urothelial, kidney renal papillary cell, and colon adenocarcinoma. High expression levels of PDLIM2 tended to be associated with higher immune and stromal scores. PDLIM2 expression was associated with the tumor mutation burden in 12 cancer types and microsatellite instability in 5 cancer types. PDLIM2 levels were strongly correlated with diverse immune-related genes. PDLIM2 can act as a prognostic-related therapeutic target and is correlated with immune infiltrates in pan-cancer.

## Introduction

With growth and aging of the global population, cancer has become the main cause of premature death in many countries and is a persistent public health challenge worldwide^1,2^. In precision medicine, immunotherapy has been applied for cancer treatment, gradually becoming a first-line treatment for many cancers, particularly in patients in advanced disease stages^3–5^. The recent introduction of immunotherapy has led to decreases in the mortality rate from tumors. However, immunotherapy shows some limitations, such as a low response rate, severe immune-related adverse events, and innate or acquired resistance to immunotherapies^6,7^. Additionally, more than half of patients do not respond to immune checkpoint inhibitor therapy^8^. Numerous studies have shown that the tumor microenvironment plays an important role in regulating the tumor immune response^9^. However, effective prognostic markers for immunotherapy are lacking. Identifying immune-related biomarkers is important for patients with cancer to provide a basis and new ideas for predicting the prognosis risk of patients.

PDLIM2^10^ is a cytoskeletal and nuclear PDZ-LIM domain protein with important roles in cytoskeleton formation, cell differentiation, signal transduction, and tumorigenesis. PDLIM2 is frequently disrupted in various cancers, and its expression is associated with both tumorigenesis and tumor suppression^11^. Mechanistically, the main function of PDLIM2 in cancer is to promote ubiquitination and proteasomal degradation of nuclear activated nuclear factor-κB RelA and STAT3 to increase the expression of genes involved in antigen presentation and T-cell activation, which leads to inhibition of malignant tumors^12,13^. A previous study^14^ showed that PDLIM2 restoration is a critical mechanism of epigenetic therapy and that PDLIM2-independent PD-L1 induction is a mechanism of acquired immune escape induced by chemotherapy and epigenetic drugs. Although PDLIM2 can inhibit some tumors, it is also highly expressed in invasive cancer cells^15^. In autoimmune diseases, PDLIM2 can restrict Th1 and Th17 differentiation^16^. PDLIM2 may be a predictive biomarker for breast cancer^17^, lung cancer^14,18^, human T-cell leukemia^19^ and colon cancer^20^. However, most studies focused on a single cancer type, and a comprehensive overview of PDLIM2 and its clinical relevance and immune infiltration in pan-cancer is lacking.

In this study, we comprehensively evaluated PDLIM2 expression and its correlation with prognosis and metastasis in patients with cancer. We further analyzed the relationship between PDLIM2 expression and tumor immune cell infiltration. We examined the usefulness of PDLIM2 as a prognostic biomarker related to tumor immune interactions and its role as a therapeutic target in combined immunotherapy in 33 cancers.

## Results

### mRNA expression levels of PDLIM2 in various human cancers

**Figure 1:**
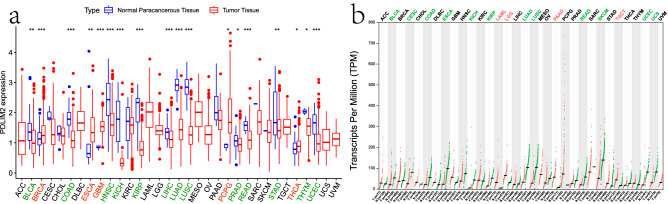
PDLIM2 expression levels in different tumor types (**a**) from TCGA database . The red fusiformis represents tumor tissue and the blue fusiformis represents normal tissue;the p values are indicated as *P < 0.05, **P < 0.01, ***P < 0.001; (**b**) from GEPIA2 database(http://gepia2.cancer-pku.cn/#analysis). Words marked in red refer to tumor types highly expressed in cancerous tissues, words marked in green refer to tumor types highly expressed in paracancerous tissues. ACC: adrenocortical carcinoma; BLCA: bladder urothelial carcinoma; COAD: colon adenocarcinoma; HNSC: head and neck squamous cell carcinoma; KICH: kidney chromophobe; KIRP: kidney renal papillary cell carcinoma; LIHC: liver hepatocellular carcinoma; LUAD: lung adenocarcinoma; LUSC: lung squamous cell carcinoma; PRAD: prostate adenocarcinoma; READ: rectum adenocarcinoma; STAD: stomach adenocarcinoma; THCA: thyroid carcinoma; UCEC: uterine corpus endometrial carcinoma; THYM: thymoma; BRCA: breast invasive carcinoma; ESCA: esophageal carcinoma; GBM: glioblastoma multiforme; PCPG: pheochromocytoma and paraganglioma (for interpretation of abbreviations in this figure legend, the reader is referred to the supplementary table of this article.)

To determine the differences in PDLIM2 expression in human cancers, we examined the RNA-sequencing data of multiple malignancies in The Cancer Genome Atlas (TCGA). The differential expression between tumor and corresponding non-tumor normal tissues for PDLIM2 across all TCGA tumors is shown in Fig. 1a. Our results indicate that PDLIM2 is more highly expressed in the normal paracancerous tissues of many cancers, compared with cancer tissues. PDLIM2 expression was significantly lower in urothelial bladder carcinoma (BLCA), colon adenocarcinoma (COAD), head and neck squamous cell carcinoma, kidney chromophobe, kidney renal papillary cell carcinoma (KIRP), liver hepatocellular carcinoma (LIHC), lung adenocarcinoma (LUAD), lung squamous cell carcinoma (LUSC), prostate adenocarcinoma, rectum adenocarcinoma, stomach adenocarcinoma, thyroid carcinoma, uterine corpus endometrial carcinoma, and thymoma than in adjacent normal tissues. PDLIM2 is repressed in most tumors, but is clearly more highly expressed in a few cancer types. Higher PDLIM2 expression was observed in breast invasive carcinoma, esophageal carcinoma, glioblastoma multiforme, pheochromocytoma, and paraganglioma.

Analysis of a GTEx dataset showed similar results (Fig. 1b). Based on these results, compared to tumor tissues, PDLIM2 is highly expressed in normal tissues from patients with BLCA, cervical squamous cell carcinoma, esophageal carcinoma, kidney chromophobe, KIRP, LUAD, LUSC, rectum adenocarcinoma, skin cutaneous melanoma, uterine corpus endometrial carcinoma, and uterine carcinosarcoma. PDLIM2 was only highly expressed in the tumor tissues of four cancer types (acute myeloid leukemia, low-grade glioma, pancreatic adenocarcinoma, and tenosynovial giant cell tumor).

### Prognostic potential of PDLIM2 in cancers

**Figure 2:**
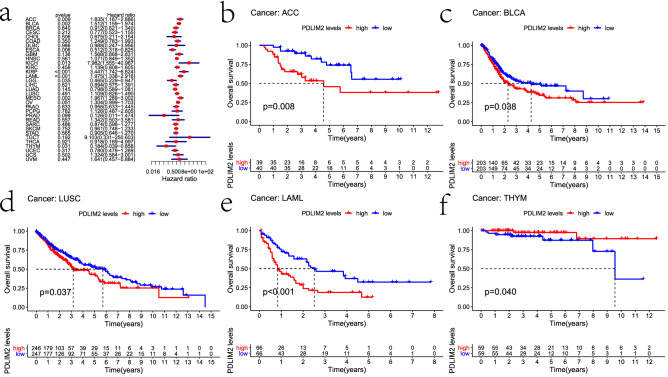
Correlation between PDLIM2 and overall survival of various cancer types of TCGA dataset by R (3.6.3 version; The R Project for Statistical Computing, Vienna, Austria). (**a**) Multivariate Cox regression analysis to identify prognosis in 33 tumors. Red squares represent the hazard ratio. (**b**–**f**) Kaplan–Meier survival curves comparing the high and low expression of PDLIM2 in different types of cancer (**b**) in ACC, (**c**) in BLCA, (**d**) in LUSC, (**e**) in LAML, (**f**) in THYM. ACC: adrenocortical carcinoma; BLCA: bladder urothelial carcinoma; LUSC: lung squamous cell carcinoma; LAML: acute myeloid leukemia; THYM: thymoma.

We investigated whether PDLIM2 expression was correlated with prognosis in patients with cancer. First, the impact of PDLIM2 expression on overall survival was analyzed using univariate survival analysis. As shown in Fig. 2a, multiple cancer types exhibited a significant association between patient prognosis and PDLIM2 expression, including breast, kidney, blood, brain, and esophageal cancer. We additionally employed the Kaplan–Meier method to assess how PDLIM2 expression is related to prognosis in various cancer types, revealing its elevation to be significantly linked with adenoid cystic carcinoma (ACC; P = 0.008), BLCA (P = 0.038), acute myeloid leukemia (P < 0.001), and LUSC (P = 0.037) (Fig. 2b–e). In contrast, reduced PDLIM2 expression was correlated with poor prognosis in thymoma (P = 0.04) (Fig. 2f).

**Figure 3:**
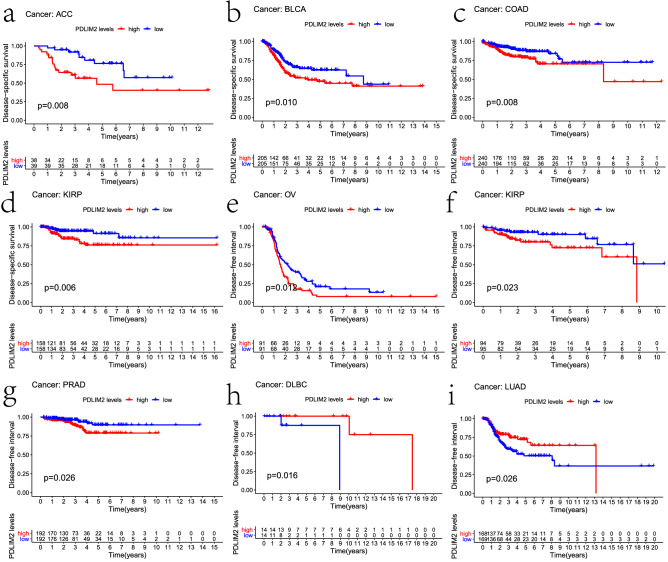
Kaplan–Meier survival curves of DSS and DFI comparing the high and low expression of PDLIM2 in different types of cancer of TCGA dataset by R (3.6.3 version; The R Project for Statistical Computing, Vienna, Austria), (**a**) DSS survival curves of ACC, (**b**) DSS survival curves of BLCA, (**c**) DSS survival curves of COAD, (**d**) DSS survival curves of KIRP, (**e**) DFI survival curves of OV, (**f**) DFI survival curves of KIRP, (**g**) DFI survival curves of PRAD, (h)DFI survival curves of DLBC, (**i**) DFI survival curves of LUAD. ACC: adrenocortical carcinoma; BLCA: bladder urothelial carcinoma; COAD: colon adenocarcinoma; KIRP: kidney renal papillary cell carcinoma; OV: ovarian serous cystadenocarcinoma; KIRP: kidney renal papillary cell carcinoma; PRAD: prostate adenocarcinoma; DLBC: lymphoid neoplasm diffuse large B-cell lymphoma; LUAD: lung adenocarcinoma.

To eliminate the influence of non-tumor-related death factors, we analyzed the relationship between gene expression and disease-specific survival. Notably, PDLIM2 expression significantly affected the prognosis of four types of cancers (Fig. 3a–d), including ACC (P = 0.004), BLCA (P = 0.01), COAD (P = 0.008), and KIRP (P = 0.006). Therefore, high PDLIM2 expression may be an independent risk factor for poor prognosis in these four types of cancers.

To further examine the prognostic potential of PDLIM2 in different cancers, we calculated the disease-free interval (DFI) for 33 types of cancer. High PDLIM2 expression levels were associated with a shorter DFI in KIRP (P = 0.023), prostate adenocarcinoma (P = 0.026), and ovarian serous cystadenocarcinoma (P = 0.012) (Fig. 3e–g). High PDLIM2 expression was also correlated with a longer DFI in lymphoid neoplasm diffuse large B-cell lymphoma (P = 0.016) and LUAD (P = 0.026) (Fig. 3h–i).

### Relationship between PDLIM2 expression and clinical stage

**Figure 4:**
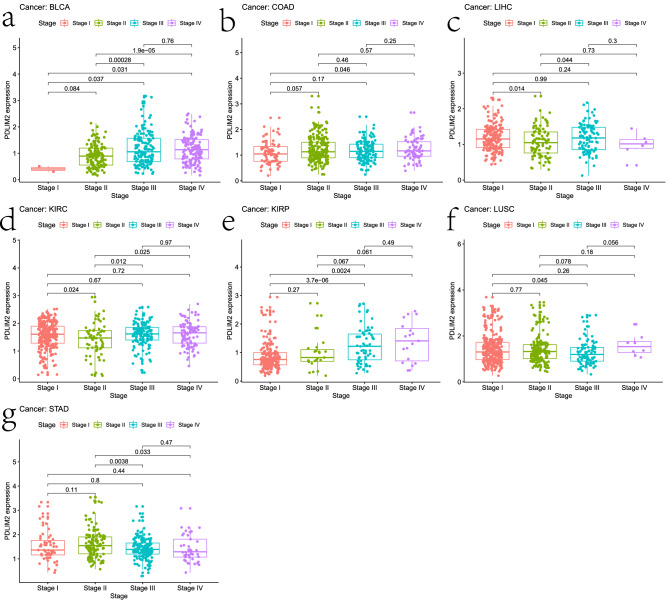
Relationship between the PDLIM2 gene expression and clinicopathological features of TCGA dataset by R (3.6.3 version; The R Project for Statistical Computing, Vienna, Austria). PDLIM2 gene expression related to the stage in BLCA (**a**), COAD (**b**), LIHC (**c**), KIRC (**d**), KIRP (**e**), LUSC (**f**) and STAD (**g**). BLCA: bladder urothelial carcinoma; COAD: colon adenocarcinoma; LIHC: liver hepatocellular carcinoma; KIRC: kidney renal clear cell carcinoma; KIRP: kidney renal papillary cell carcinoma; LUSC: lung squamous cell carcinoma; STAD: stomach adenocarcinoma.

We evaluated the relationship between PDLIM2 expression and the clinical stage of 33 cancer types. In this analysis, PDLIM2 expression was significantly correlated with the tumor stage in seven types of cancers. Specifically, high PDLIM2 mRNA expression was positively correlated with tumor stage in BLCA, kidney renal clear cell carcinoma, LIHC, KIRP, COAD, and LUSC (Fig. 4a–f). In contrast, patients with stage 3 gastric cancer showed lower PDLIM2 expression than patients with stage 2 disease (Fig. 4g). These results suggest that PDLIM2 expression influences the prognosis of patients with gastrointestinal, lung, liver, and renal cancer by affecting lymph node metastasis. PDLIM2 expression was higher in the highest-grade tumors than in low-grade tumors, suggesting that PDLIM2 is involved in promoting cancer progression or metastasis.

### Correlation between PDLIM2 expression and immune cell infiltration

**Figure 5:**
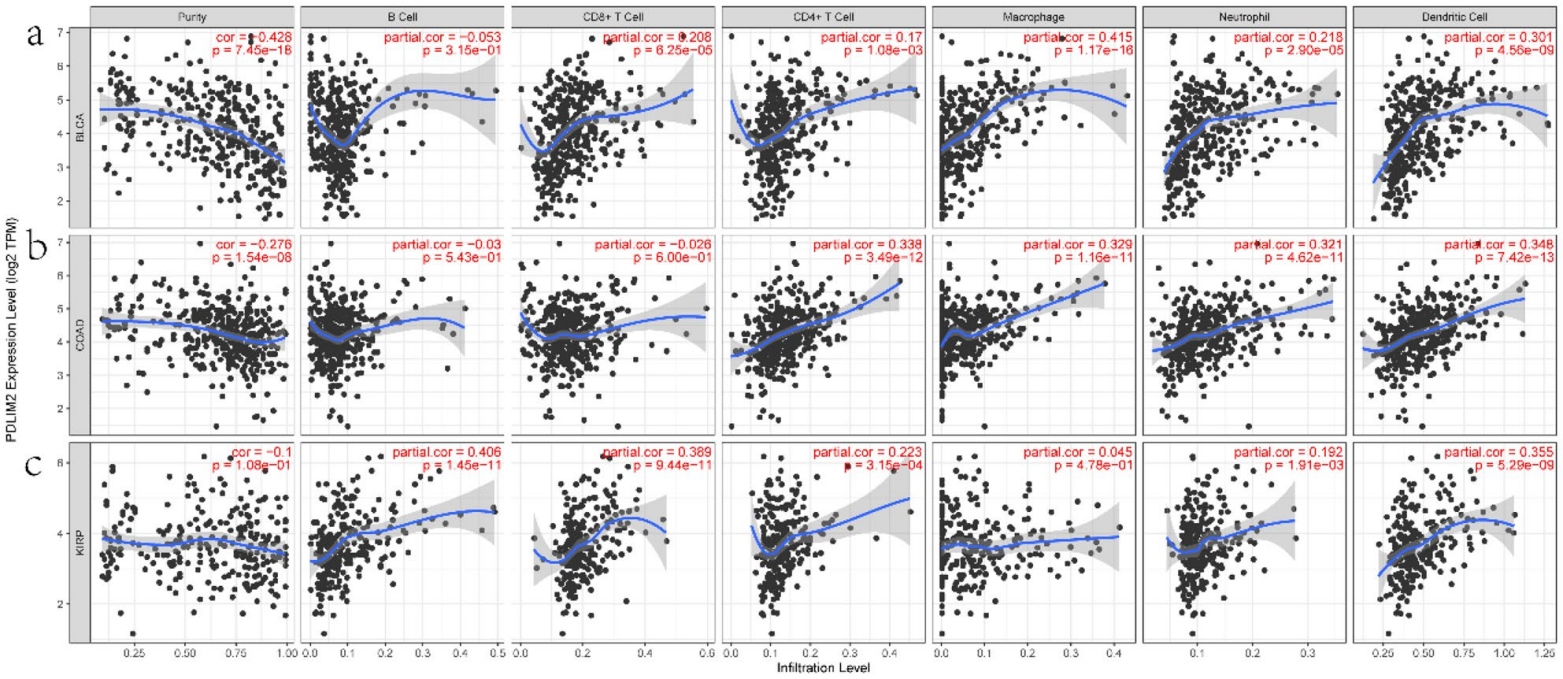
PDLIM2 expression level has significant positive correlations with immune infiltration level by TIMER database(https://cistrome.shinyapps.io/timer/) in BLCA, COAD and KIRP, (**a**) in BLCA, (**b**) in COAD, (**c**) in KIRP. BLCA: bladder urothelial carcinoma; COAD: colon adenocarcinoma; KIRP: kidney renal papillary cell carcinoma.

Immune cell infiltration affects survival and tumor metastasis in patients. In the previous analysis, PDLIM2 expression levels were found to be associated with prognosis and metastasis in BLCA, KIRP, and COAD. The correlation between the expression level of PDLIM2 and the six types of infiltrating immune cells in these three tumor types is shown in Fig. 5. PDLIM2 expression was negatively correlated with tumor purity. Tumor purity refers to the proportion of cancer cells in tumor samples. Tumors with low tumor purity tend to have a higher mutational load and stronger immune phenotypes^21,22^. The expression level of PDLIM2 was positively correlated with macrophages (R = 0.415, P = 1.17e−16) and dendritic cells (R = 0.301, P = 4.56e−09) in BLCA; CD4+ T cells (R = 0.338, P = 3.49e−12), macrophages (R = 0.329,P = 1.16e−11), neutrophils (R = 0.321, P = 4.62e−11), and dendritic cells (R = 0.348, P = 7.42e−13) in COAD; and B cells (R = 0.406, P = 1.45e−11), CD8+ T cells (R = 0.389, P = 9.44e−11), and dendritic cells (R = 0.355, P = 5.29e−09) in KIRP.

### Correlation between PDLIM2 expression level and immune cell markers

High PDLIM2 expression was positively correlated with immune cell infiltration. We further investigated the correlation between PDLIM2 expression levels and immunological marker sets in BLCA, COAD, and KIRP (Table 1). The results revealed that the PDLIM2 expression level was positively correlated with the expression of most immune cell markers in these three cancers, particularly in BLCA and KIRP. Based on this result, the expression levels of most marker sets of monocytes, tumor-associated macrophages (TAMs), M2 macrophages, dendritic cells, and T cell exhaustion were positively correlated with PDLIM2 expression. Specifically, PDLIM2 expression was correlated with monocyte markers (CD86, CSF1R), TAM markers (CCL2, IL-10), and M2 markers (CD163, VSIG4, MS4A4A) in BLCA, suggesting that PDLIM2 regulates macrophage differentiation and leads to poor prognosis. A similar relationship was observed for KIRP. In addition, PDLIM2 showed a significant relationship with dendritic cell markers (HLA-DPB1, HLA-DQB1, HLA-DRA, HLA-DPA1) in BLCA and KIRP, indicating that PDLIM2 is closely related to the infiltration of dendritic cells. There was a marked correlation between the expression of PDLIM2 and marker genes of Treg and T cell exhaustion, particularly in KIRP, which may lead to immune dysfunction and poor prognosis in patients with cancer, confirming the role of PDLIM2 in tumor immune escape.

**Table 1:** Correlation analysis between PDLIM2 and relate genes and markers of immune cells of TCGA dataset by R (3.6.3 version; The R Project for Statistical Computing, Vienna, Austria) (the P values are indicated as *P < 0.05, **P < 0.01, ***P < 0.001).

Description	Gene markers	BLCA		COAD		KIRP	
		Cor	P-value	Cor	P-value	Cor	P-value
CD8+ T cell	CD8A	0.234	***	0.207	***	0.418	***
	CD8B	0.256	***	0.122	**	0.419	***
T cell (general)	CD3D	0.293	***	0.300	***	0.454	***
	CD3E	0.314	***	0.274	***	0.440	***
	CD2	0.313	***	0.173	***	0.409	***
B cell	CD19	0.315	***	0.185	***	0.191	**
	CD79A	0.353	***	0.329	***	0.386	***
Monocyte	CD86	0.400	***	0.202	***	0.313	***
	CSF1R	0.493	***	0.451	***	0.340	***
TAM	CCL2	0.425	***	0.201	***	0.173	**
	CD68	0.215	***	0.021	0.655	0.112	0.056
	IL10	0.430	***	0.185	***	0.309	***
M1 Macrophage	NOS2	0.039	0.428	0.053	0.248	0.153	**
	IRF5	0.084	0.087	0.287	***	0.067	0.260
	PTGS2	0.049	0.319	0.009	0.841	0.090	0.126
M2 Macrophage	CD163	0.497	***	0.288	***	0.289	***
	VSIG4	0.482	***	0.379	***	0.319	***
	MS4A4A	0.476	***	0.243	***	0.281	***
Neutrophils	CEACAM8	0.062	0.211	0.042	0.361	0.042	0.475
	ITGAM	0.406	***	0.402	***	0.273	***
Natural killer cell	KIR2DL1	0.078	0.117	0.029	0.532	0.206	***
	KIR2DL3	0.069	0.165	0.022	0.631	0.182	**
	KIR2DL4	0.092	0.064	0.043	0.350	0.327	***
	KIR3DL1	0.077	0.120	0.034	0.459	0.139	*
	KIR3DL2	0.059	0.230	0.114	*	0.274	***
	KIR3DL3	0.066	0.179	0.020	0.671	0.061	0.299
	KIR2DS4	0.075	0.127	0.003	0.942	0.227	***
Dendritic cell	HLA-DPB1	0.408	***	0.417	***	0.365	***
	HLA-DQB1	0.348	***	0.261	***	0.340	***
	HLA-DRA	0.330	***	0.281	***	0.358	***
	HLA-DPA1	0.340	***	0.310	***	0.354	***
	NRP1	0.259	***	0.172	***	0.022	0.714
	CD1C	0.251	***	0.276	***	0.112	0.058
	ITGAX	0.442	***	0.332	***	0.198	***
Th1	TBX21	0.256	***	0.135	**	0.353	***
	STAT4	0.284	***	0.004	0.937	0.259	***
	STAT1	0.129	**	0.027	0.554	0.068	0.251
	IFNG	0.157	**	0.032	0.489	0.312	***
	TNF	0.062	0.209	0.191	***	0.050	0.396
Th2	GATA3	0.268	***	0.352	***	0.282	***
	STAT6	0.189	***	0.232	***	0.177	**
	STAT5A	0.256	***	0.245	***	0.100	0.091
	IL13	0.079	0.109	0.182	***	0.047	0.422
Tfh	BCL6	0.257	***	0.153	***	0.046	0.434
	IL21	0.126	*	0.031	0.504	0.012	0.839
Th17	STAT3	0.125	*	0.017	0.706	0.001	0.988
	IL17A	0.049	0.322	0.085	0.064	0.105	0.076
Treg	FOXP3	0.315	***	0.380	***	0.399	***
	CCR8	0.164	***	0.183	***	0.300	***
	STAT5B	0.043	0.380	0.083	0.070	0.201	***
T cell exhaustion	TGFB1	0.363	***	0.530	***	0.398	***
	PDCD1	0.292	***	0.281	***	0.366	***
	CTLA4	0.290	***	0.140	**	0.367	***
	LAG3	0.269	***	0.242	***	0.437	***
	HAVCR2	0.421	***	0.251	***	0.162	**
	GZMB	0.313	***	0.050	0.283	0.473	***

### Correlation between PDLIM2 expression level and tumor microenvironment

**Figure 6:**
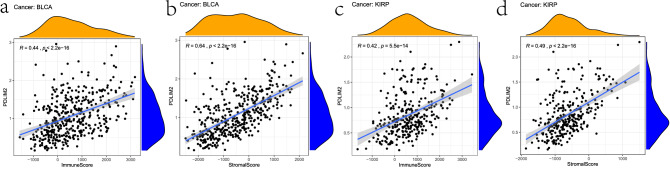
Correlation between PDLIM2 and immuneScore and Stromal Score of TCGA dataset by R (3.6.3 version; The R Project for Statistical Computing, Vienna, Austria). in BLCA and KIRP, (**a**) correlation between PDLIM2 and immuneScore in BLCA, (**b**) correlation between PDLIM2 and Stromal Score in BLCA, (**c**) correlation between PDLIM2 and immuneScore in KIRP, (**d**) Correlation between PDLIM2 and Stromal Score in KIRP; BLCA: bladder urothelial carcinoma; KIRP: kidney renal papillary cell carcinoma.

The expression of PDLIM2 may be closely related to immune escape in BLCA and KIRP. The immune score predicts the response to tumor immunotherapy. Analysis of the relationship between PDLIM2 expression and immune scores revealed that higher PDLIM2 expression levels were associated with higher immune scores and stromal scores (Fig. 6).

We next examined the relationship between the expression of immune checkpoint genes (including 11 immunostimulators and 7 immunoinhibitors) and PDLIM2. The expression of PDLIM2 was significantly associated with that of immune checkpoint genes (Table 2).

**Table 2:** Correlation analysis between PDLIM2 and immune checkpoint genes in BLCA and KIRP of TCGA dataset by R (3.6.3 version; The R Project for Statistical Computing, Vienna, Austria).

Type	Gene name	BLCA		KIRP	
		Cor	P-value	Cor	P-value
Immunostimulators	CD27	0.339225	1.59E−12	0.436264	7.40E−15
	ICOSLG	0.071695	0.146802	0.239827	3.79E−05
	TNFRSF14	− 0.02662	0.590558	− 0.06828	0.247237
	TNFRSF18	0.283498	4.91E−09	0.245397	2.46E−05
	TNFRSF25	− 0.20447	2.96E−05	0.0574	0.330863
	TNFRSF4	0.441261	5.18E−21	0.458971	1.83E−16
	TNFRSF8	0.421468	3.95E−19	0.4159	1.62E−13
	TNFRSF9	0.333119	4.16E−12	0.290495	5.01E−07
	TNFSF14	0.222245	5.39E−06	0.162223	0.005707
	TNFSF15	− 0.13163	0.007539	0.137494	0.019368
	TNFSF4	0.530316	3.47E−31	0.121316	0.039299
Immunoinhibitors	ADORA2A	0.139962	0.004472	0.341575	2.49E−09
	CD160	− 0.0164	0.740333	0.019327	0.743544
	HAVCR2	0.420765	4.59E−19	0.162168	0.005723
	LGALS9	0.072353	0.143117	0.29105	4.76E−07
	PDCD1LG2	0.385971	4.75E−16	0.477368	7.44E−18
	TIGIT	0.246545	4.16E−07	0.458281	2.06E−16
	VTCN1	0.030564	0.536652	− 0.1394	0.017732

### Correlation between PDLIM2 expression level and tumor mutational burden and microsatellite instability

**Figure 7:**
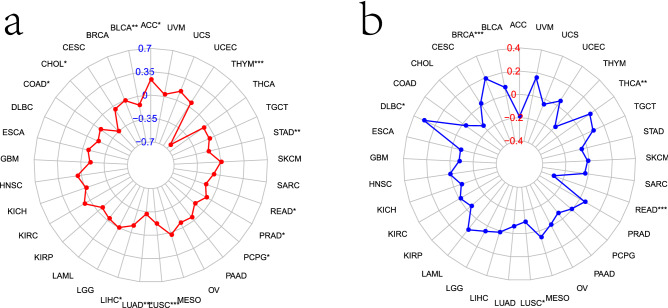
Relationships between PDLIM2 gene expression and TMB, MSI of TCGA dataset in Pan-cancer by R (3.6.3 version; The R Project for Statistical Computing, Vienna, Austria). (**a**) The radar chart illustrated the association between PDLIM2 and TMB in 33 cancer types. The red curve represents the correlation coefficient, and the blue value represents the range. (**b**) The radar chart illustrated the relationship between PDLIM2 and MSI in 33 cancer types. The blue curve represents the correlation coefficient, and the green value represents the range. TMB, tumor mutational burden; MSI, microsatellite instability; *P < 0.05, **P < 0.01, and ***P < 0.001. ACC: adrenocortical carcinoma; BLCA: bladder urothelial carcinoma; COAD: colon adenocarcinoma; HNSC: head and neck squamous cell carcinoma; KICH: kidney chromophobe; KIRP: kidney renal papillary cell carcinoma; LIHC: liver hepatocellular carcinoma; LUAD: lung adenocarcinoma; LUSC: lung squamous cell carcinoma; PRAD: prostate adenocarcinoma; READ: rectum adenocarcinoma; STAD: stomach adenocarcinoma; THCA: thyroid carcinoma; UCEC: uterine corpus endometrial carcinoma; THYM: thymoma; BRCA: breast invasive carcinoma; ESCA: esophageal carcinoma; GBM: glioblastoma multiforme; PCPG: pheochromocytoma and paraganglioma (for interpretation of abbreviations in this figure legend, the reader is referred to the supplementary table of this article).

Previous studies focused on the prognostic role of tumor mutational burden (TMB) in immunotherapy in many cancer types; here, we counted the TMB of each tumor sample and analyzed the relationship between PDLIM2 expression and TMB in 12 different cancer types. PDLIM2 was negatively correlated with the TMB in BLCA, cholangiocarcinoma, COAD, LIHC, LUAD, LUSC, paraganglioma, prostate adenocarcinoma, pulmonary enteric adenocarcinoma, stomach adenocarcinoma, and thymoma and positively correlated with ACC (Fig. 7a).

The microsatellite instability (MSI) status of different solid tumors was significantly different from that of checkpoint inhibitor drugs for the immune response rates. Several studies have shown that MSI is closely related to tumor occurrence. Analysis of the correlation between PDLIM2 expression and MSI revealed a correlation mainly in adenocarcinoma (Fig. 7b). PDLIM2 expression was positively correlated with MSI in breast invasive carcinoma, diffuse large B-cell lymphoma, and thyroid carcinoma. In contrast, MSI was negatively correlated with LUSC and rectal adenocarcinoma.

### Correlation between PDLIM2 expression level and immune checkpoint genes

**Figure 8:**
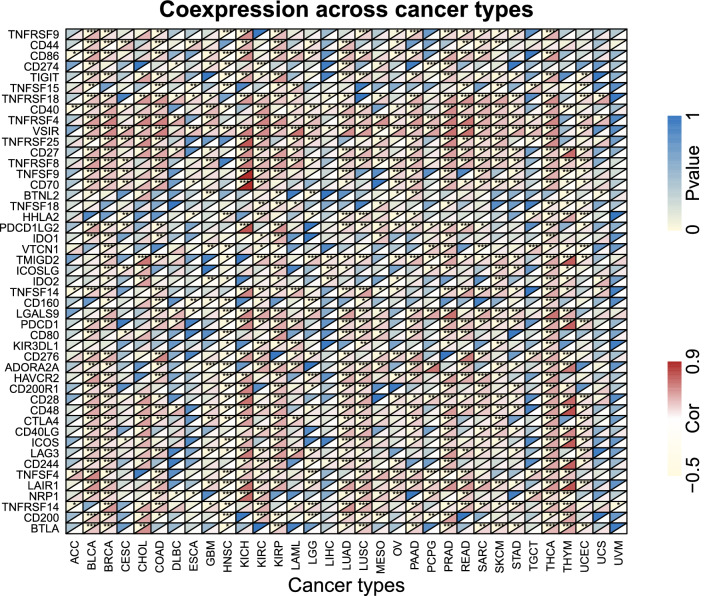
Correlation analysis between PDLIM2 and immune checkpoint genes in 33 cancer types of TCGA dataset by R (3.6.3 version; The R Project for Statistical Computing, Vienna, Austria). The horizontal axis represents cancer types, the vertical axis represents immune genes, each small rectangular module represents the co-expression of immune gene and PDLIM2 gene in cancer, during them, upper left corner asterisk and color represent the P-value, and the lower right corner color represents the Cor. *P < 0.05; **P < 0.01; ***P < 0.001. Cor, correlation coefficient. ACC: adrenocortical carcinoma; BLCA: bladder urothelial carcinoma; COAD: colon adenocarcinoma; HNSC: head and neck squamous cell carcinoma; KICH: kidney chromophobe; KIRP: kidney renal papillary cell carcinoma; LIHC: liver hepatocellular carcinoma; LUAD: lung adenocarcinoma; LUSC: lung squamous cell carcinoma; PRAD: prostate adenocarcinoma; READ: rectum adenocarcinoma; STAD: stomach adenocarcinoma; THCA: thyroid carcinoma; UCEC: uterine corpus endometrial carcinoma; THYM: thymoma; BRCA: breast invasive carcinoma; ESCA: esophageal carcinoma; GBM: glioblastoma multiforme; PCPG: pheochromocytoma and paraganglioma (for interpretation of abbreviations in this figure legend, the reader is referred to the supplementary table of this article).

Immune checkpoints are signaling pathways responsible for downregulating the immune response to avoid destruction of endogenous targets and temper the peripheral immune response. We analyzed the relationship between the expression of common immune checkpoint genes and PDLIM2 in 33 cancer types. The results showed that PDLIM2 expression was significantly associated with immune checkpoint expression in most tumor types, although there was no significant relationship in ACC, esophageal carcinoma, LIHC, uterine carcinosarcoma, and uveal melanoma (Fig. 8). Notably, PDLIM2 was related not only to the common targets of checkpoint inhibitors (PDCD1, CTLA4, etc.), but also to targets of stimulatory checkpoint molecules (CD27, ICOS, etc.).

## Discussion

PDLIM2 is a member of the PDZ-LIM family, whose PDZ structural domain is a functional module for protein–protein inter-recognition and the LIM structural domain is a structural domain for protein–protein interactions. These structures play important roles in cell differentiation and signal transduction^23^. PDLIM2 is crucial for tumorigenesis and progression and is closely related to the immune system. We comprehensively characterized PDLIM2 across 33 cancer types and highlight the potential clinical utility of immunity therapy for PDLIM2 expression.

The expression of PDLIM2 is related to both tumor inhibition and tumorigenesis^11^. Our results support the dual role of PDLIM2 in cancer. PDLIM2 is a putative tumor-suppressor protein that is inhibited by epigenetics in different cancers and is highly expressed in most adjacent noncancerous tissues. PDLIM2, as a cytoskeleton component, can reverse the growth of cancer cells by regulating promoter methylation. Repression of PDLIM2 has been shown to continuously activate nuclear factor-κB and STAT3, eventually leading to tumorigenesis and tumor maintenance^24^. Sun et al. found that PDLIM2 is inhibited in lung cancer, which is associated with a poor prognosis^14^. However, in our study, PDLIM2 led to different prognoses in different types of lung cancer. As seen by clinical correlation analysis, the reason for this discrepancy may be that high PDLIM2 expression contributes to the poor prognosis of LUSC patients by affecting lymph node metastasis. In addition, PDLIM2 was also highly expressed in invasive cancer cells. Zhao et al. reported that PDLIM2 promotes ovarian cancer growth in vivo and in vitro through NOS2-derived nitric oxide signaling^25^. Another study showed that PDLIM2 inhibition effectively reduced the tumor growth and invasiveness of human castration-resistant prostate cancer cells^26^. A recent study revealed that PDLIM2 was highly correlated with tumor growth and metastasis in renal cell carcinoma in a mouse knockout model^27^. We also observed significant correlations between PDLIM2 expression and patient survival in thymic carcinomas, which has not been widely reported in previous studies. Additionally, gene expression was related to clinical stage in seven tumors, suggesting that PDLIM2 is involved in promoting cancer progression or metastasis. 1,25 (OH) 2D3-induced adhesion of cancer cells to the extracellular matrix is mediated by PDLIM2^28^. PDLIM2 promotes tumor angiogenesis by activating the MAPK/ERK pathway^26^ depending on the tumor microenvironment^29^. These results confirm the prognostic value of PDLIM2 in some specific types of cancers and that increased and decreased PDLIM2 expression have different prognostic values depending on the cancer type.

Importantly, we found that PDLIM2 functions in the recruitment and activation of immune infiltrating cells. PDLIM2 participates in the differentiation of TAMs into M2 macrophages; TIMER database results showed positively correlated with the infiltration of B cells and macrophages in BLCA and KIRP. We showed that PDLIM2 expression was not related to M1 macrophages but was positively correlated with most M2 macrophage markers. A previous study revealed that PDLIM2 leads to the recruitment of M2 macrophages in ovarian cancer^25^. TAMs lead to poor prognosis of tumors by inhibiting the immune and secreting various factors that promote tumor growth^30^. M2 macrophages can promote tumors growth and secrete IL-10, transforming growth factor β, and other mediators that contribute to establishing a tumor-tolerant microenvironment and angiogenic factors^31^. IL-10 (a TAM marker) is often associated with decreased T-cell activation^32^. In addition, PDLIM2 may inhibit T cell-mediated immunity and is involved in the immune escape of tumors. The correlation between PDLIM2 expression and immune cell marker genes suggests that PDLIM2 can control the infiltration and interaction of immune cells in the tumor microenvironment, and Treg-like immunosuppression can be induced by inducing Foxp3 expression in naïve T cells^33^. Dendritic cells can lead to tumor metastasis by enhancing the Treg response and suppressing the cytotoxicity of CD8+ T cells^34^. Notably, PDLIM2 expression was strongly correlated with TIM-3 in bladder cancer and with PDCD1, CTLA4, GZMB, and LAG3 in KIRP. These genes are common markers for T cell exhaustion, which is one of the main factors of immune dysfunction in patients with cancer. PD-1-or CTL-4-mediated pathways suppress T cell function^35^. LAG3 can inhibit T cells without relying on CD4^36^. T cell exhaustion can be partially reversed clinically using immune checkpoint inhibitors^37^. Patients with high PDLIM2 expression showed higher T cell infiltration; thus, these patients are more likely to benefit from immune checkpoint blockade against PD-1 and CTLA-4. Generally, PDLIM2 can mediate the differentiation of M2 macrophages and T cell exhaustion, thus avoiding immune detection.

Another key finding of this study is that PDLIM2 can be used as a therapeutic target for epigenetic drugs combined with immune checkpoint inhibitors. Patients with high expression benefit more from immunotherapy. PDLIM2 expression was associated with the TMB in 12 cancer types and MSI in five cancer types. TMB^38–40^ and MSI are important in the immunotherapy response. The TMB is a pan-cancer genomic biomarker related to the efficacy of checkpoint inhibitors. A higher TMB is an easier target for tumor immunotherapy. MSI^41^ is also an indicator of the efficacy of immunotherapy and was first used in colon cancer. Interestingly, although MSI indicators are more suitable for digestive system tumors, there is no correlation between PDLIM2 and MSI. PDLIM2 is a potential target for combination therapy for cancer. Notably, the expression of PDLIM2 was significantly associated with immune checkpoint expression in most tumor types. Guo and Qu suggested that PDLIM2 can be used as an epigenetic drug with immunomodulatory potential in combined immunotherapy for cancer^42^. Administration of epigenetic drugs can also enhance the efficacy of immunological checkpoint treatments^43^.

There were some limitations to this study. First, all analyses were performed using public datasets. Our findings should be verified in animal experiments and hospitalized patients. Second, although we found that the expression of PDLIM2 is associated with tumor immune cell infiltration and survival, we did not confirm that PDLIM2 affects the survival of patients through immune infiltration, and the prognostic value of this protein in tumor immune mechanisms and immune signals should be further explored. Finally, we only included total PDLIM2 RNA levels in this study, without considering RNA variants and protein modifications.

In summary, PDLIM2 can affect pan-cancer prognosis and participate in immune regulation. Particularly, in BLCA and KIRP, PDLIM2 is mainly associated with immunosuppression in tumor tissues. Therefore, PDLIM2 is useful as a prognostic-related biomarker and is correlated with immune infiltrates in the BLCA and KIRP. Moreover, PDLIM2 may be a valuable therapeutic target for tumor immunotherapy in 33 cancer types.

## Methods

### Data acquisition

A total of 479 datasets of gene expression profiles, mutation data, and clinical information from TCGA^44^ was downloaded from UCSC Xena^45^ (http://xena.ucsc.edu/). Among them, only survival data were downloaded from the clinical data. To ensure statistical power, we also downloaded curated clinical information from TCGA Pan-CANCER (pan can) including 41 datasets and 12,591 samples to obtain data on disease-specific survival and DFI. After eliminating cases with insufficient or missing data for age and the overall survival time, 11,057 samples were included in the study (Supplementary Table S2).

### Gene expression analysis

We used “wilcox.test” to analyze the differential expression of total PDLIM2 RNA in normal and tumor tissue samples from 33 cancer types in TCGA database and drew a box plot using “ggpubr.”

To compensate for the lack of normal organization in TCGA database, we added the GTEx(Genotype-Tissue Expression) database^46^ for analysis; this part of the analysis was mainly performed in the Gene Expression Profile of the GEPIA2 database^47^ (Gene Expression Profiling Interactive Analysis, http://gepia2.cancer-pku.cn/#analysis). The main parameters were as follows: gene, *PDLIM2*; differential methods, analysis of variance; q-value cutoff, 0.05; matched normal data: match TCGA normal and GTEx data.

### Survival clinical analysis

Univariate survival analysis was performed using the Kaplan–Meier survival” package^48^. According to the median PDLIM2 expression level, patients with cancer were divided into low- and high-expression groups. Cox regression analysis was performed only for overall survival (OS). Kaplan–Meier analysis was conducted to compare the survival (OS, DDS, and DFI) differences between the low- and high-expression groups. A P value less than 0.05 was considered as the threshold in the Kaplan–Meier analysis results. The “survival” package was used for survival analysis. We used the R package “forestplot” to draw the forest map in Cox survival analysis and R package “survivminer” to draw the Kaplan–Meier survival curve.

A stage plot using the tumor stage as a variable was plotted to analyze the relationship between the expression level of PDLIM2 and tumor metastasis in different cancers. R package “limma”^49^ was used to analyze the differential expression of PDLIM2 in different clinical stages, and the box diagram was drawn using “ggpubr.”

### TIMER database analysis

TIMER^50^ (Tumor Immune Estimation Resource, https://cistrome.shinyapps.io/timer/) can use RNA-sequencing expression profile data to detect the infiltration of immune cells in tumor tissue. We applied the Gene of Immune Association module to explore the correlation between PDLIM2 expression and the abundance of immune infiltrates in BLCA, KIRP, and COAD. We imported “PDLIM2” in gene symbol; selected BLCA, KIRP, and COAD for cancer types; and chose B cells, CD4+ T cells, neutrophils, CD8+ T cells, macrophages, and dendritic cells for immune infiltrates.

### Mutation analysis

TMB is a useful biomarker for predicting the prognosis and efficacy of immunotherapy. We calculated the TMB of the 33 tumor types using mutation data. Perl scripts were written to extract genomic alterations, and calculate the TMB of the patients.TMB refers to the number of somatic mutations per million bases in tumor tissue^51^. MSI values were derived from TCGA database. We then analyzed the correlation between PDLIM2, TMB, and MSI and designed a radar map using the R-package “fmsb.” Correlation analysis was performed using Spearman’s correlation.

### Immunological correlation analysis

In the tumor microenvironment, immune cells and stromal cells are two main types of non-tumor components and were suggested to be valuable in the diagnosis and prognosis of tumors. We used R package “estimate”^52^ to calculate the immune score and stromal score for BLCA and KIRP. Correlation analysis was performed using Spearman’s correlation. The correlation between PDLIM2 and the immune score and stromal score was analyzed and plotted using “ggplot2,” “ggpubr,” and “ggextra.”

### Co-expression analysis

We evaluated common immune checkpoint and immune marker genes. The co-expression relationship between PDLIM2 and these genes was calculated using limma package. A heat map of PDLIM2 co-expression with immune checkpoint genes was drawn using the R packages “reshape2” and “RColorBrewer.” All graphics and data analyses were completed on the R platform (3.6.3 version; The R Project for Statistical Computing, Vienna, Austria).

### Statement

All methods were carried out in accordance with relevant guidelines and regulations.

## Supplementary Information

Supplementary Tables.

## Data Availability

The datasets obtained from public database.
