# Supplementary material for: Systematic evaluation of the prognostic and immunological role of PDLIM2 across 33 cancer types
Source: Sci Rep. 2022 Feb 4;12:1933. doi: 10.1038/s41598-022-05987-1 (PMC8817018; doi:10.1038/s41598-022-05987-1)
Supplement: Supplementary file 1 — Supplementary Tables. [file 41598_2022_5987_MOESM1_ESM.docx]

**Systematic evaluation of the prognostic and immunological role of PDLIM2 across 33 cancer types**

Yudan Zeng ^1^, Dongtao Lin^1^, Mengqian Gao^1^, Guoxia Du ^1^, Yongming Cai^2,3,*^

^1^ School of Public Health, Guangdong Pharmaceutical University, Guangzhou, China

^2^ College of Medical information Engineering, Guangdong Pharmaceutical University, Guangzhou, China

^3^ Guangdong Provincial TCM Precision Medicine Big Data Engineering Technology Research Center, Guangzhou, China

^*^ Corresponding author

Supplementary table 1: Supplementary table Abbreviation of this paper

| Abbreviation | Full name |
| --- | --- |
| TCGA | The Cancer Genome Atlas |
| GTEx | Genotype-Tissue Expression |
| GEPIA2 | Gene Expression Profiling Interactive Analysis |
| TIMER | Tumor Immune Estimation Resource |
| OS | overall survival |
| TMB | Tumor mutation burden |
| DSS | Disease-specific survival |
| PFI | Progression-free interval |
| MSI | DNA microsatellite instability |
| ACC | Adrenocortical carcinoma |
| BLCA | Bladder Urothelial Carcinoma |
| BRCA | Breast invasive carcinoma |
| CESC | Cervical squamous cell carcinoma and endocervical adenocarcinoma |
| CHOL | Cholangiocarcinoma |
| COAD | Colon adenocarcinoma |
| DLBC | Lymphoid Neoplasm Diffuse Large B-cell Lymphoma |
| ESCA | Esophageal carcinoma |
| GBM | Glioblastoma multiforme |
| HNSC | Head and Neck squamous cell carcinoma |
| KICH | Kidney Chromophobe |
| KIRC | Kidney renal clear cell carcinoma |
| KIRP | Kidney renal papillary cell carcinoma |
| LAML | Acute Myeloid Leukemia |
| LGG | Brain Lower Grade Glioma |
| LIHC | Liver hepatocellular carcinoma |
| LUAD | Lung adenocarcinoma |
| LUSC | Lung squamous cell carcinoma |
| MESO | Mesothelioma |
| OV | Ovarian serous cystadenocarcinoma |
| PAAD | Pancreatic adenocarcinoma |
| PCPG | Pheochromocytoma and Paraganglioma |
| PRAD | Prostate adenocarcinoma |
| READ | Rectum adenocarcinoma |
| SARC | Sarcoma |
| SKCM | Skin Cutaneous Melanoma |
| STAD | Stomach adenocarcinoma |
| TGCT | Testicular Germ Cell Tumors |
| THCA | Thyroid carcinoma |
| THYM | Thymoma |
| UCEC | Uterine Corpus Endometrial Carcinoma |
| UCS | Uterine Carcinosarcoma |
| UVM | Uveal Melanoma |

Supplementary table 2: Number of tumor and normal cases about 33 cancers of TCGA datebase

| Cancer type | Tumor tissue | Paracancerous tissue | Total |
| --- | --- | --- | --- |
| ACC | 79 | 0 | 79 |
| BLCA | 411 | 19 | 430 |
| BRCA | 1104 | 113 | 1217 |
| CESC | 306 | 3 | 309 |
| CHOL | 36 | 9 | 45 |
| COAD | 471 | 41 | 512 |
| DLBC | 48 | 0 | 48 |
| ESCA | 162 | 11 | 173 |
| GBM | 168 | 5 | 173 |
| HNSC | 502 | 44 | 546 |
| KICH | 65 | 24 | 89 |
| KIRC | 535 | 72 | 607 |
| KIRP | 289 | 32 | 321 |
| LAML | 151 | 0 | 151 |
| LGG | 529 | 0 | 529 |
| LIHC | 374 | 50 | 424 |
| LUAD | 526 | 59 | 585 |
| LUSC | 501 | 49 | 550 |
| MESO | 86 | 0 | 86 |
| OV | 379 | 0 | 379 |
| PAAD | 178 | 4 | 182 |
| PCPG | 183 | 3 | 186 |
| PRAD | 499 | 52 | 551 |
| READ | 167 | 10 | 177 |
| SARC | 263 | 2 | 265 |
| SKCM | 471 | 1 | 472 |
| STAD | 375 | 32 | 407 |
| TGCT | 156 | 0 | 156 |
| THCA | 510 | 58 | 568 |
| THYM | 119 | 2 | 121 |
| UCEC | 548 | 35 | 583 |
| UCS | 56 | 0 | 56 |
| UVM | 80 | 0 | 80 |
| Total | 10327 | 730 | 11057 |
